# Hookworm Disease

**Published:** 1911-01

**Authors:** 


**Hookworm Disease.** By **George Dock,** A.M., M.D., Professor of the Theory and Practice of Medicine, Medical Department Tulane University of Louisiana, New Orleans, and **Charles C. Bass, M.D.,** Instructor of Clinical Microscopy and Clinical Medicine in the same university. Royal octavo, 250 pages. Illustrated. St. Louis: C. V. Mosby Company, Publishers. (Price, $2.50.)

During the past two or three years the subject of hookworm disease has attracted widespread attention, particularly in that part of the country in which it is most prevalent. In this book the subject is exhaustively treated, and by men who know the disease well and have had a large experience in its treatment. From Virginia south to Florida and Texas, according to the writers of the book, the disease is very common, and among certain employees of cotton mills almost universal, as many as 80 per cent. of the workers being hookworm bearers. A knowledge of this subject is of such vast importance to the southern medical man that this work should meet with a large demand. J. A. R.

